# Open-Window Thoracostomy Closure Using a Free Musculocutaneous Flap, Fascia Patch Graft, and Postoperative Compression Guided by Near-Infrared Spectroscopy: A Case Report

**DOI:** 10.3390/jcm15124574

**Published:** 2026-06-12

**Authors:** Paloma Malagón, Cristian Carrasco, Carlos Martinez-Barenys, Sebastián Peñafiel, Martin Marzabal, Linda Klimavicius Palma, Carmen Higueras

**Affiliations:** 1 Department of Plastic Surgery, Hospital Germans Trias i Pujol, Universitat Autònoma de Barcelona, 08916 Barcelona, Spain; carralo@gmail.com (C.C.); martinmarzabal@gmail.com (M.M.); 2 Department of Thoracic Surgery, Hospital Germans Trias i Pujol, 08916 Barcelona, Spain; cmartinezb.germanstrias@gencat.cat (C.M.-B.); spenafielg.germanstrias@gencat.cat (S.P.); lklimaviciusp.germanstrias@gencat.cat (L.K.P.); 3 Chief of the Department of Plastic Surgery, Hospital Germans Trias i Pujol, Universitat Autònoma de Barcelona, 08916 Barcelona, Spain; drahiguerascpl@gmail.com

**Keywords:** open-window thoracostomy, free flap, postoperative monitoring, compression, near-infrared spectroscopy

## Abstract

Bronchopleural fistula is a rare but severe complication of lung resection, associated with significant morbidity and mortality, especially when an open-window thoracostomy is required. The clinical and surgical management is complex and becomes even more challenging in the presence of underlying conditions such as recurrent infections or malignancy. Postoperative management is equally demanding, as local compression may help prevent fistula recurrence but can compromise flap perfusion. A 65-year-old male with a history of right upper lobectomy and subsequent sublobar resection for lung adenocarcinoma presented with an 8 × 4 cm open-window thoracostomy complicated by chronic bronchopleural fistula and empyema. Extensive fibrosis of the surrounding tissues, including the ipsilateral latissimus dorsi muscle, limited the available reconstructive locoregional options. Reconstruction was performed using primary fistula closure reinforced with a contralateral free latissimus dorsi musculocutaneous flap and a fascia patch graft secured with cyanoacrylate-based bioadhesive. Postoperatively, continuous near-infrared spectroscopy monitoring enabled safe application of compressive bandage while minimizing the risk of flap perfusion compromise. Complete fistula closure was achieved. Apart from a surgical site abscess requiring debridement on postoperative day 7, no further complications occurred. At the 2-year follow-up, the patient remains free of fistula recurrence, wound dehiscence, or oncological relapse. We describe a novel approach for open-window thoracostomy closure combining a free musculocutaneous flap with a fascia patch graft reinforced by bioadhesive, together with postoperative perfusion monitoring using near-infrared spectroscopy. This strategy may help address both the reconstructive and postoperative challenges associated with complex bronchopleural fistulas.

## 1. Introduction

Bronchopleural fistula is a rare but serious complication after a lobectomy or a pneumonectomy with a high risk of morbidity and mortality, especially when an open-window thoracostomy is required [1]. Its diagnosis and assessment may be challenging and often require multiple imaging evaluations together with invasive tests such as bronchoscopy, thoracoscopy, or open surgical exploration [2]. In clinical practice, the diagnosis is not limited to confirming the existence of a fistula; it also requires defining the size, location, and associated pleural contamination in order to select the most appropriate reconstructive strategy.

Its clinical and surgical management is highly complex and becomes even more challenging in the presence of underlying conditions such as recurrent infections or malignancy [3]. Bronchopleural fistula associated with empyema represents one of the most demanding reconstructive scenarios in thoracic surgery due to the combination of impaired tissue vascularization, dead space, persistent infection, and the need to achieve a durable airtight closure [1,3]. In this context, treatment failure is rarely related to a single factor; rather, it usually reflects the interaction between poor tissue quality, chronic inflammatory changes, and the mechanical stress generated by respiration and coughing.

Successful treatment mainly depends on two key factors: secure closure of the bronchopleural fistula and adequate obliteration of the cavity using well-vascularized tissue [4]. Various surgical strategies, including local and free flap reconstruction as well as bronchoscopic stenting, have been described with variable outcomes [1,5]. However, these approaches are not universally applicable and must be individualized according to the characteristics of the defect, the condition of the surrounding tissues, and the availability of recipient vessels. For large or irregular postresection cavities, especially in previously irradiated fields, free tissue transfer may offer the best reconstructive option because it provides healthy vascularized tissue and permits multilayer closure of complex defects.

Several local and regional flap options have been described for intrathoracic and chest wall reconstruction, including muscle, musculocutaneous, and omental flaps [6,7,8,9,10]. However, in patients with chronic bronchopleural fistula and empyema, reconstructive management is frequently complicated by repeated thoracic procedures, chronic inflammation, persistent infection, radiotherapy, and extensive fibrosis. These factors may severely compromise the availability and reliability of local and regional tissues, especially in the presence of dense scarring or partial muscle loss. In such situations, free tissue transfer may represent the most reliable reconstructive option, as it provides a large volume of healthy, well-vascularized tissue capable of obliterating dead space, improving local vascularity, and promoting infection control and wound healing.

Despite successful reconstruction, postoperative management remains challenging. External compression is often recommended during the immediate postoperative period to reduce dead space, limit air leakage, and minimize the risk of recurrent fistula formation, especially during coughing or Valsalva maneuvers. However, excessive compression may compromise flap perfusion, especially in microsurgical reconstruction, where even transient vascular impairment can compromise flap viability. This creates a clinically relevant dilemma between achieving adequate mechanical stabilization of the thoracic repair and preserving optimal blood flow within the flap.

To our knowledge, this is the first case report that combines a musculocutaneous free flap with fascia patch graft reinforced with bioadhesives for the closure of an open-window thoracostomy, and monitoring with non-invasive near-infrared spectroscopy for applying controlled postoperative local compression. By integrating microsurgical reconstruction, multilayer fistula reinforcement, and continuous postoperative perfusion monitoring, this approach aims to address both the technical challenges of achieving durable airtight closure and the complexities associated with postoperative management in these high-risk patients.

## 2. Case Report

A 65-year-old male was referred to our department with an 8 × 4 cm right open-window thoracostomy. Two years earlier, he had undergone a right upper lobectomy followed by adjuvant chemotherapy for lung adenocarcinoma (pT3N0M0). The following year, a relapse occurred and was treated with a sublobar resection of the right lower lobe. The case was complicated by a persistent bronchopleural fistula and empyema that required an open-window thoracostomy (Figure 1 and Figure 2). In addition, there were recurrent infections and an episode of hemoptysis that was treated with embolization of the bronchial artery. The chronicity of the disease, together with the oncologic background, repeated thoracic interventions, and persistent inflammatory process, resulted in severely compromised local tissues with extensive fibrosis, distorted anatomy, and limited reconstructive options. From a reconstructive standpoint, the case represented a particularly challenging scenario due to the need to achieve a durable airtight closure while simultaneously obliterating the residual intrathoracic cavity in a previously operated and chronically infected field.

Preoperative assessment included chest CT angiography and ultrasound evaluation of the thoracic wall and potential donor sites to identify available recipient vessels and assess local tissue quality. Both imaging tests revealed no suitable perforators near the thoracostomy and significant scarring of the local tissue. A major portion of the latissimus dorsi muscle had been resected or was fibrotic, while the thoracodorsal pedicle remained intact in the proximal part. These findings supported the indication for free tissue transfer rather than a local or regional reconstruction.

The procedure was performed under general anesthesia using a simultaneous two-team approach with the patient in the prone position. This approach allowed simultaneous preparation of the recipient and donor sites, thereby reducing operative time and avoiding intraoperative repositioning, which could have increased both anesthetic complexity and perioperative morbidity in a patient with compromised respiratory reserve.

The thoracic surgery team performed extensive local debridement of chronically inflamed and devitalized tissues, followed by careful exploration of the pleural cavity and identification of the bronchopleural fistula. Three distinct points of air leakage were identified and primarily repaired using interrupted 3-0 Vicryl sutures reinforced with pledgets to improve the mechanical stability of the closure.

Simultaneously, the plastic surgery team harvested a contralateral latissimus dorsi musculocutaneous free flap together with a separated fascial graft from the left side. The dimensions of the skin paddle were tailored according to the size of the thoracostomy defect and cavity requirements. The graft was used to reinforce the fistula closure and fixed with cyanoacrylate-based adhesive (Figure 3A). The addition of the fascial patch provided an extra mechanical barrier between the bronchial closure and the transferred flap, with the aim of reinforcing airtight sealing in a setting associated with a high risk of recurrence and persistent air leakage.

Recipient vessels were the right thoracodorsal vessels, which were dissected intramuscularly to perform the anastomosis closer to the defect and facilitate a tension-free flap insetting for obliterating the cavity (Figure 3B). End-to-end anastomoses were performed using interrupted 8-0 nylon sutures for the artery and the COUPLER system for the vein. Flap and local tissue perfusions were additionally assessed intraoperatively using indocyanine green angiography. Meticulous debridement of residual fibrotic and non-viable tissue was also carried out to optimize the local environment and improve the likelihood of durable healing.

The flap was carefully adapted to completely obliterate the residual cavity and provide multilayer coverage of the thoracostomy. The skin paddle was secured with 2-0 Vicryl and 3-0 Monocryl (Figure 3C). Closed-suction drains were placed at both donor and recipient sites. The sensor of the near-infrared spectroscopy (INVOS System, Medtronic; Dublin, Ireland) was attached over the skin paddle, and a compressive dressing was applied.

## 3. Postoperative Management

During the immediate postoperative period, a compressive bandage was maintained, and flap perfusion was monitored using the near-infrared spectroscopy system. This setup allowed continuous postoperative monitoring of tissue oxygenation while maintaining the local compressive force considered necessary to reduce dead space, minimize air leakage, and decrease the risk of recurrent fistula formation. Whenever a significant decrease in tissue oxygen saturation was detected [11], immediate positional changes and adjustments of the compressive dressing were performed to relieve excessive local pressure (Figure 4). This dynamic postoperative management strategy allowed real-time adaptation of the degree of compression according to flap perfusion status, thereby reducing the risk of ischemic compromise while preserving the beneficial mechanical effect of compression on the thoracic repair.

## 4. Follow-Up

On postoperative day 7, the patient required a second surgical procedure for drainage and debridement of a localized abscess at the recipient site. Despite this complication, the free flap remained completely viable, with no evidence of arterial or venous insufficiency, partial necrosis, or wound dehiscence.

Near-infrared spectroscopy monitoring allowed early identification of significant decreases in flap oxygen saturation associated with patient positioning and compressive dressing adjustments, all of which resolved immediately after repositioning. Following abscess debridement, the postoperative course was otherwise uneventful, with progressive healing and no evidence of persistent air leakage, recurrent empyema, or respiratory deterioration.

A scheduled follow-up CT scan for lung cancer monitoring showed no oncological recurrence and a complete closure of the fistula (Figure 5A). The patient remains clinically free of dehiscence or fistula recurrence two years after surgery (Figure 5B,C).

## 5. Discussion

Open-window thoracostomy due to chronic bronchopleural fistula and empyema is a life-threatening condition and represents one of the most challenging scenarios in thoracic and reconstructive surgery. The main difficulties are achieving a durable airtight closure of the fistula while simultaneously obliterating the residual cavity and controlling local infection. In many patients, this complexity is further increased by chronic inflammation, repeated surgical procedures, distorted anatomy, and extensive fibrosis, which frequently compromise the quality and availability of local tissues for reconstruction.

In addition, previous studies have reported considerable morbidity and relatively high in-hospital mortality associated with bronchopleural fistula closure [12]. For this reason, management requires not only technical closure of the defect itself, but also careful surgical planning and close postoperative monitoring. From a reconstructive perspective, factors such as respiratory motion, coughing, persistent dead space, and the risk of recurrent infection or air leakage must all be anticipated during both surgical design and postoperative care.

Several techniques have been described to reinforce bronchopleural fistula closure, including the use of muscle flaps and fascial patch grafts, with the aim of reducing the risk of dehiscence and recurrence [12,13]. Thoracic wall reconstruction can be achieved using a variety of locoregional flaps, including the latissimus dorsi, pectoralis major, serratus anterior, intercostal muscle, and omental flaps [14,15,16,17]. However, in this case, repeated infections, multiple previous interventions, and chronic fistula formation had resulted in extensive fibrosis and damage to the surrounding tissues. As a result, the ipsilateral latissimus dorsi and other local pedicle flaps were considered unsuitable. In addition, preoperative CTA and ultrasound evaluation confirmed the absence of suitable perforators near the defect, while the thoracodorsal vessels remained patent and of good quality. For these reasons, a free flap was considered the most reliable reconstructive option.

The ideal free flap for this case needed to provide sufficient bulk to obliterate the irregular cavity, a reliable vascular pedicle of adequate length to reach the thoracodorsal vessels, and the tissue components necessary to achieve multilayer closure. The contralateral latissimus dorsi musculocutaneous flap fulfilled all of these requirements. Its large size and pliability allowed effective obliteration of the cavity, while the combination of muscle and skin paddle facilitated multilayer reconstruction. In addition, the flap could be harvested simultaneously with fistula repair in the prone position, avoiding patient repositioning and reducing overall operative time.

Despite performing free flap reconstruction together with multilayer closure of the defect, achieving and maintaining a completely airtight seal remains difficult in these patients. Persistent air leakage may lead to recurrent fistula formation, chronic infection, and failure of the reconstruction. In addition, even minor postoperative bleeding may become a potentially life-threatening complication if blood enters the airway through an incompletely sealed bronchopleural communication during the early postoperative period. For this reason, a fascial patch graft was interposed between the lung and the flap and reinforced with cyanoacrylate-based adhesive in an attempt to improve airtight sealing and provide an additional protective barrier. Beyond reinforcing the bronchial repair itself, this layer was intended to reduce direct communication between the airway and the reconstructed cavity in the event of early partial dehiscence or recurrent leakage.

From a reconstructive perspective, this multilayer approach offers several potential advantages. By separating the bronchial repair from the free flap, the fascial patch may reduce direct mechanical stress on the suture line caused by respiratory motion, coughing, or pressure changes within the thoracic cavity. At the same time, it creates an additional interface between the airway and the transferred tissue, potentially contributing to both mechanical stability and safer postoperative healing in a chronically inflamed and previously infected environment.

Postoperative management represented another major challenge in this case. External compression is commonly recommended after bronchopleural fistula closure in order to reduce dead space, minimize air leakage, and decrease the risk of recurrence, particularly during coughing or Valsalva maneuvers, which are frequent in these patients. However, excessive compression may compromise flap perfusion, especially in microsurgical reconstruction, where even transient vascular impairment can threaten flap viability and negatively affect wound healing. Previous studies have shown that several postoperative factors, including external pressure and patient positioning, may significantly influence tissue perfusion after free flap reconstruction [18]. In the present case, attention was therefore paid to maintaining an adequate balance between mechanical stabilization of the thoracic repair and preservation of microvascular perfusion within the transferred flap. Because postoperative compression was considered important to reduce the risk of recurrent air leakage, continuous flap monitoring was performed using near-infrared spectroscopy. This technique provides a non-invasive, objective, and continuous assessment of tissue oxygenation and has previously demonstrated high sensitivity for detecting early perfusion changes in free flap reconstruction [11,19,20,21,22,23,24,25].

In our experience, near-infrared spectroscopy proved especially useful during the immediate postoperative period, as it enabled rapid detection of transient decreases in flap oxygen saturation related to patient positioning and compressive dressing adjustments. In this patient, NIRS facilitated the postoperative management of a potentially conflicting reconstructive requirement: the need for local compression to support fistula closure while preserving flap viability. Continuous oxygen saturation monitoring allowed the application of compressive dressings with immediate feedback on tissue perfusion. All of the decreased oxygen saturations were promptly identified and corrected through adjustment of patient positioning and dressing pressure, resulting in immediate recovery of perfusion parameters (Figure 4). This allowed postoperative compression to be maintained with greater confidence while preserving flap perfusion, which was especially important in a patient with a high risk of recurrent air leakage and frequent coughing episodes. Although no conclusions regarding superiority over standard clinical monitoring can be drawn from a single case, this experience suggests that NIRS may represent a useful adjunct in complex reconstructive scenarios where flap assessment is limited by compressive dressings, reduced flap visibility, or challenging postoperative positioning.

A wound complication occurred on the seventh postoperative day, requiring surgical debridement of an abscess. However, the fistula repair remained intact, with no evidence of dehiscence or recurrence. Given the history of chronic infection, empyema, multiple prior thoracic procedures, and extensive fibrosis, this complication was considered more likely related to the compromised local tissue environment than to failure of the reconstructive technique. Durable fistula closure was maintained throughout follow-up without further postoperative complications.

Despite the need for reintervention due to a postoperative abscess, complete fistula closure was achieved without further postoperative complications or recurrence during follow-up. The flap remained fully viable throughout the postoperative course, and no episodes of persistent air leakage, wound dehiscence, or recurrent empyema were observed. From a reconstructive perspective, the favorable long-term outcome suggests that the combination of controlled postoperative compression and continuous tissue oxygenation monitoring may be helpful in selected high-risk patients in whom both dead-space management and preservation of microvascular perfusion are critical.

Although this report describes a single case, the present approach combines several complementary strategies aimed at addressing different aspects of a challenging reconstructive problem: reinforcement of the bronchial repair using a fascial patch graft and bioadhesive, obliteration of the cavity with well-vascularized free tissue, and postoperative compression guided by continuous perfusion monitoring. In patients with chronic infection, extensive fibrosis, and limited local reconstructive options, this multimodal strategy may represent a useful alternative for achieving stable closure while reducing the risk of recurrence. Nevertheless, additional studies including larger series and longer follow-up are necessary to better assess reproducibility, complication rates, functional outcomes, and long-term reliability of this approach.

## 6. Conclusions

This case demonstrates a possible reconstructive strategy for the management of a complex bronchopleural fistula in the setting of an open-window thoracostomy. The combination of a free musculocutaneous flap, fascia patch graft, and postoperative compression monitored by NIRS allowed successful fistula closure and flap surveillance in this patient. Further experience is required to determine the reproducibility and potential role of this approach in similar cases.

## Figures and Tables

**Figure 1:**
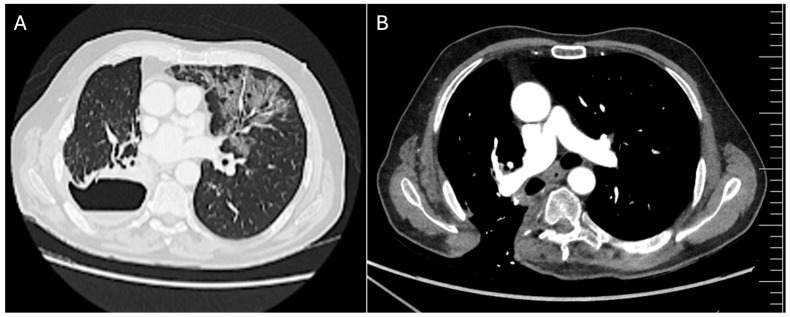
(**A**) Chest CT scan following lung adenocarcinoma resection that shows a right bronchopleural fistula with a hydroaeric level and infectious involvement of the contralateral lung. (**B**) Chest CT scan showing the right open-window thoracostomy performed for the management of the persistent fistula.

**Figure 2:**
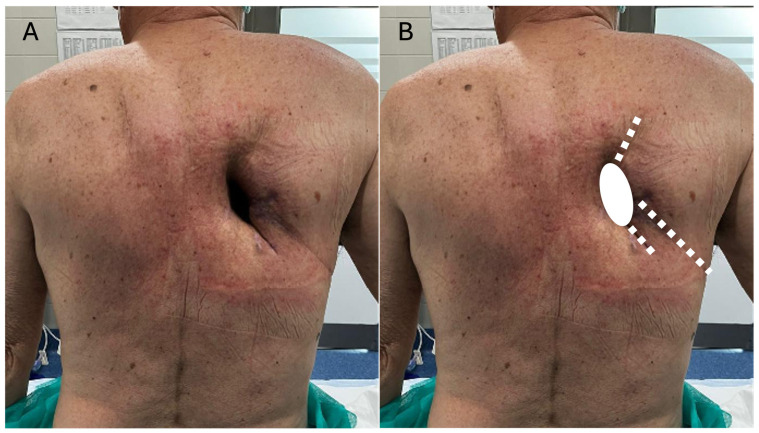
(**A**). Preoperative photograph of the open-window thoracostomy. (**B**). Right open-window thoracostomy and scars (white oval) and scars of previous surgeries (white lines), with one of them over the latissimus dorsi muscle.

**Figure 3:**
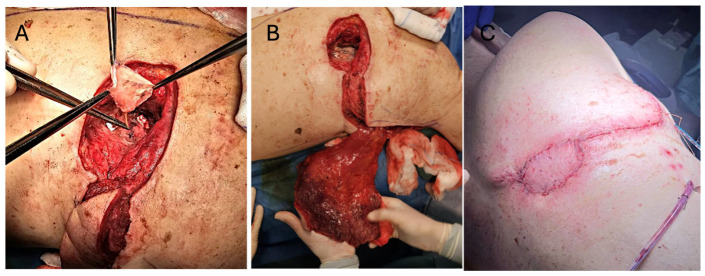
Intraoperative images of the fascia patch graft (**A**), musculocutaneous latissimus dorsi free flap anastomosed to the thoracodorsal vessels (**B**), and the immediate postoperative result (**C**).

**Figure 4:**
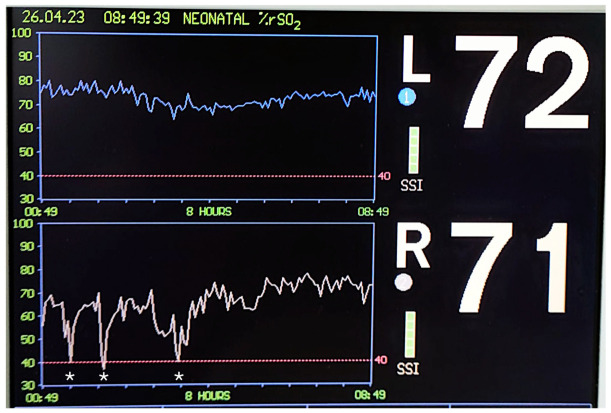
Postoperative monitoring of the oxygen saturation during the last 8 h using near-infrared spectroscopy. Two sensors were used: sensor 1 was used as a control (above) and sensor 2 was placed on the paddle of the latissimus dorsi flap (below). There were 3 events (*) with a significant drop in the oxygen saturation of the flap, which were immediately solved with positional changes of the patients and adjustments of the compression bandage.

**Figure 5:**
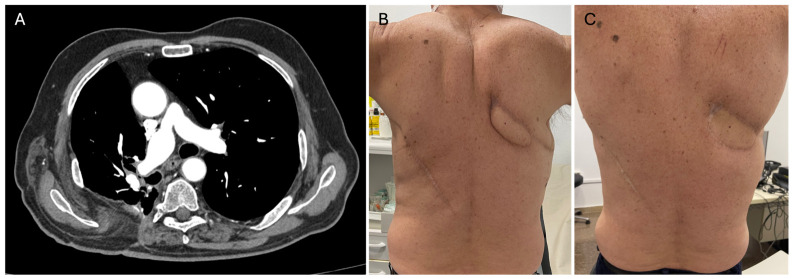
Postoperative CT scan that shows the flap covering the open-window thoracostomy, and no signs of bronchopleural or pleurocutaneous fistulas, residual cavities, infection or malignancy (**A**). Clinical outcomes at 9 months (**B**) and 19 months (**C**) after surgery, with no recurrence of fistula or infections.

## Data Availability

The data presented in this study are not publicly available due to patient privacy and ethical restrictions. Data are available from the corresponding author upon reasonable request.
